# Occupational accidents involving biological material during dental instrument cleaning: a Brazilian national study

**DOI:** 10.1590/1807-3107bor-2026.vol40.035

**Published:** 2026-06-22

**Authors:** Anaclara Ferreira Veiga Tipple, Júnnia Pires de Amorim Trindade, Gileade Pereira Freitas, Rafael Alves Guimarães

**Affiliations:** (a) Universidade Federal de Goiás – UFG, School of Nursing, Goiânia, GO, Brazil.; (b) Universidade Federal de Goiás – UFG, School of Dentistry, Goiânia, GO, Brazil.

**Keywords:** Epidemiology, Occupational Injuries, Occupational Risks, Biohazard Release, Dentistry

## Abstract

Despite evidence regarding the severity of occupational accidents involving biological material (OAIBM) during dental instrument cleaning, these risks remain underestimated by many workers. Few studies, however, have focused on the epidemiology of OAIBM during dental instrument cleaning. Thus, this study aimed to identify, analyze, and estimate the incidence rate of OAIBM during dental instrument cleaning by dental care professionals in Brazil. Notifications registered between January 2015 and July 2020 were then extracted from the Notifiable Diseases Information System (SINAN), a Brazilian government agency responsible for reporting and investigating infectious diseases. Reports of OAIBM were analyzed by dental care professionals (dentists, oral health assistants [OHA], and oral health technicians [OHT]) from all 26 Brazilian states and the Federal District. The mean incidence rate of OAIBM among dental care professionals in Brazil during dental instrument cleaning was 314.5 cases per 100,000 professionals. Of these cases, 88.2% involved OHTs, and most affected individuals were female (94.4%). Serum, blood, or plasma accounted for most exposures (71.8%), and 85.1% of exposures were percutaneous. Gloves were the most commonly used personal protective equipment (PPE) at the time of the accidents. Therefore, this study highlights the role of the dentist as the leader and technical authority, responsible for providing resources, standardizing processes, training the team, and supervising performance to ensure occupational safety, quality processing, and contributions to patient safety.

## Introduction

Occupational risks are present in any work environment. They can cause harm to workers in various forms, including ergonomic, physical, chemical, and biological hazards, the latter of which is the most frequent among health professionals.^1^

Several pathogens (viruses, bacteria, fungi, prions, and protozoa) can be transmitted during occupational exposure.^2^ The most epidemiologically significant agents are the human immunodeficiency virus, hepatitis B virus, and hepatitis C virus, with the risk of transmission occurring most frequently through occupational accidents involving sharp instruments.^3^

The World Health Organization (WHO) has estimated that, among 35 million healthcare professionals worldwide, two to three million are at risk of percutaneous exposure to bloodborne pathogens annually.^4^ Among these professionals, the highest prevalence of accidents occurs among dental care professionals (59.1%), most frequently involving anesthesia needles.^5^

Among the strategies for preventing occupational exposure, the use of personal protective equipment (PPE) represents the primary control measure. PPE is defined as any device used individually by workers to protect against risks to their health and promote safety in the workplace. Therefore, PPE is considered essential for protecting workers, regardless of their area of activity.^6,7^

The processing of health care materials involves several steps, including cleaning, drying, preparation, packaging, sterilization, and storage. Of these steps, cleaning is considered the most important for successful processing of health care products.^8,9^ Nevertheless, cleaning also poses the highest occupational risk because it involves repeated and intensive handling of contaminated materials.^10^ Despite evidence demonstrating the severity of occupational accidents involving biological material (OAIBM) during instruments cleaning and recommending the use of PPE as the most effective preventive measure, the literature shows many professionals still underestimate such risks.^11–13^ PPE recommended for instrument cleaning includes heavy and long-cuff gloves (latex, nitrile, or butyl), a waterproof long-sleeved gown, protective goggles, a mask, a cap, and closed waterproof shoes.^8^

Despite the importance of OAIBM during dental instrument cleaning, few studies have addressed its epidemiology. Therefore, this study aimed to identify, analyze, and estimate the incidence rate of OAIBM during dental instrument cleaning by dental care professionals in Brazil.

## Methodology

This was a retrospective analytical cohort study. Data were extracted for analysis from the Notifiable Diseases Information System (SINAN), a Brazilian government agency responsible for infectious disease reporting and investigation. Access to the database was granted by the Workers’ Health Coordination of the Health Surveillance Secretariat, Brazilian Ministry of Health. SINAN data originate from mandatory reporting and investigation forms, which consist of standardized records containing sociodemographic, occupational, and clinical information from both public and private institutions.^14^

The study included reports of OAIBM by dental care professionals (dentists, oral health assistants [OHAs], and oral health technicians [OHTs]) during dental instrument cleaning across the 26 Brazilian states and the Federal District. Notifications registered between January 2015 and July 2020 were analyzed. Incidence rates were calculated using data from the National Registry of Health Establishments (CNES), which provides information on the number of health professionals working in Brazil. Accidents involving exposure to biological material were defined as occupational events resulting in direct or indirect exposure to biological material potentially contaminated by pathogens (e.g., viruses, bacteria, fungi, prions, and protozoa) through sharp or non-sharp instruments, according to the International Classification of Diseases (ICD) - code Z20.9.^15–17^ Dental instrument cleaning was defined as the process whereby organic and inorganic debris are removed and the microbial load present on the instruments is reduced, using water, detergents, cleaning products, and accessories through manual or automated cleaning processes.^18^

Data were analyzed using STATA software (version 16.0). The Descriptive analysis was used to characterize the study population. Variables were described as absolute (n) and relative (%) frequencies. The incidence rate of OAIBM during dental instrument was estimated using the following formula:


$$\text{Incidence rate}=\frac{\text{Number of accidents during dental instrument cleaning}}{\text{Number of health professionals}}\times 100000$$

The incidence rate was estimated for Brazil, major geographic regions, states, and the Federal District. Inferential analyses included bivariate and multivariable analyses and were performed to identify OAIBM-associated factors during dental instrument cleaning.

Bivariate Poisson regression was performed to examine associations between the dependent variable and each independent variable. Variables with a p-value <0.20 were included in a multivariate Poisson regression model with robust variance using the single-entry method. Multicollinearity was assessed using a correlation matrix, with Pearson correlation coefficients ≥ 0.6 considered to be collinear. Bivariate analysis results were presented as unadjusted relative risk (RR) and corresponding 95% confidence intervals (95% CI). Multiple regression results were expressed as adjusted relative risk (aRR), regression coefficients (β), and 95% CI. Quality of the final model adjustment was assessed by the goodness-of-fit test, with statistical significance determined by the Wald test. McFadden's coefficient (McFadden's R2) was used to assess the model's explanatory power. In all analyses, p-values < 0.05 were considered statistically significant.

## Results

Between January 2015 and July 2020, a total of 324,173 cases of OAIBM were reported in Brazil. Among these, 17,407 involved dental care professionals. Of these cases, 14,853 (85.3%) did not occur during dental instrument cleaning, whereas 2,554 (14.7%) occurred during the cleaning process.

The mean age of professionals involved in OAIBM during dental instrument cleaning was 33.2 years (±10.2), with most individuals aged 18 to 29 years (42.2%) and 30 to 39 years (33.4%). Most affected individuals were female (94.4%), followed by dental assistants (70.0%) and public servants (43.8%). The year 2018 recorded the highest number of notifications (21.1%), whereas 2020 had the lowest number of notifications (5.8%). Serum, blood, or plasma was the most frequently reported biological material (71.8%), with percutaneous exposure occurring in 85.1% of cases, but 59.6% of the accidents involved other agents. At the time of the accident, glove use was reported in 92.8% of cases (type not specified as examination gloves or surgical gloves), followed by gowns (71.6%), protective goggles (37.6%), masks (50.5%), and boots (14.2%). Most dental care professionals (90.4%) reported complete vaccination against hepatitis B virus (Table 1).

**Table 1:** Clinical, demographic, and occupational accident characteristics of dental care professionals during dental instrument cleaning. Brazil, 2015-2020.

Variables		Total (n = 17,407)		No (n = 14,853)		Yes (n = 2,554)	
		n	%	n	%	n	%
Age (years), mean (SD)		33.7 (10.6)		33.8 (10.6)		33.2 (10.2)	
Age range (years)							
	18–29	7,654	44.0	6,575	44.3	1,079	42.2
	30–39	5,14	29.5	4,286	28.9	854	33.4
	40–49	2,875	16.5	2,471	16.6	404	15.9
	50–59	1,423	8.2	1,233	8.3	190	7.4
	> 60	315	1.8	288	1.9	27	1.1
Sex							
	Male	3,016	17.3	2,874	19.3	142	5.6
	Female	14,391	82.7	11,979	80.7	2,412	94.4
Region							
	Southeast	7,574	43.5	6,596	44.4	978	38.3
	Northeast	3,247	18.7	2,781	18.7	466	18.2
	Central	1,821	10.5	1,538	10.4	283	11.1
	South	3,753	21.6	3,06	20.6	693	27.2
	North	1,012	5.8	878	5.9	134	5.2
Education							
	Higher education	10,311	59.2	10,009	67.4	302	11.8
	Technical education	7,096	40.8	4,844	32.6	2,252	88.2
Professional education							
	Dentist	10,311	59.2	10,009	67.4	302	11.8
	OHA	5,573	32.0	3,786	25.5	1,787	70.0
	OHT	1,523	8.7	1,058	7.1	465	18.2
Employment status							
	EFC	4,281	29.8	3,43	28.2	851	38.5
	Civil servant	5,53	38.4	4,564	37.5	966	43.8
	Others	4,577	31.8	4,186	34.3	391	17.7
	Missing data	3.019		2.673		346	
Year of notification							
	2015	2,823	16.2	2,388	16.1	435	17.0
	2016	2,945	16.9	2,521	17.0	424	16.6
	2017	3,377	19.4	2,908	19.6	469	18.5
	2018	3,514	20.2	2,974	20.0	540	21.1
	2019	3,686	21.2	2,149	21.2	537	21.0
Types of exposure							
	Percutaneous	14,352	83.2	12,196	82.9	2,156	85.1
	Missing data	156		135		21	
	Intact skin	4,962	28.8	4,23	28.7	732	28.9
	Missing data	156		135		21	
	Non-intact skin	776	4.5	665	4.5	111	4.4
	Missing data	156		135		21	
	Oral/Ocular mucosa	961	5.6	917	6.2	44	1.7
	Missing data	156		135		21	
Biological material							
	Serum/blood/plasma	13,581	83.6	11,968	85.5	1,613	71.8
	Cavity fluids	97	0.6	82	0.6	15	0.7
	Blood-containing fluid	1,401	8.6	1,094	7.8	307	13.7
	Other fluids	1,165	7.2	852	6.1	313	13.8
	Missing data	1,163		857		306	
Instrument							
	Needle	10,112	59.8	9,364	65.0	748	30.1
	Blade	1,141	6.8	886	6.1	255	10.3
	Others	5,643	33.4	4,161	28.9	1,482	59.6
	Missing data	511		442		69	
Use of PPE							
	Gloves	15,963	94.3	13,382	94.6	2,311	92.8
	Missing data	765		700		65	
	Gown	12,741	79.1	11,013	80.4	1,728	71.6
	Missing data	1,29		1,151		826	
	Protective goggles	9,771	61.0	8,875	65.1	896	37.6
	Missing data	1,391		1,217		174	
	Mask	11,96	74.4	10,752	78.6	1,208	50.5
	Missing data	1,329		1,165		164	
	Boots	2,191	14.4	1,863	14.5	328	14.2
	Missing data	2,207		1,961		246	
Vaccination against HBV							
	Yes	14,503	92.6	12,42	93.0	2,083	90.4
	No	1,161	7.4	940	7.0	221	9.6
	Missing data	1,743		1,493		250	

SD: standard deviation; OHA: oral health assistant; OHT: oral health technician; EFC: Employee with a formal contract; PPE: personal protective equipment; HBV: hepatitis B virus.

The incidence of OAIBM during dental instrument cleaning by dental care professionals in Brazil averaged 314.5 cases per 100,000 professionals. Tocantins was the Brazilian state with the highest incidence rate (752.4 cases per 100,000 professionals), whereas Amazonas showed the lowest rate (115.0 cases per 100,000 professionals). The incidence of OAIBM during dental instrument cleaning by dentists in Brazil averaged 39.1 cases per 100,000 dentists. No cases were reported for the states of Amapá and Piauí, while Tocantins had the highest average incidence rate (106.2 cases per 100,000 dentists). Among OHAs and OHTs in Brazil, the incidence of OAIBM during dental instrument cleaning averaged 5,686.2 cases per 100,000 professionals. The lowest incidence rate was observed in Amazonas (801.7 cases per 100,000 professionals), whereas the highest rate was recorded for Tocantins (19,699.8 cases per 100,000 professionals) (Table 2).

**Table 2:** Average rate of occupational accidents involving biological material during cleaning stage of dental instruments in dentistry by Brazilian Federative Unit (every 100,000 professionals). Brazil, 2015-2020.

Brazilian states and Federal District	Dental care professionals	Dentist	OHA/OHT
Amazonas	115.0	22.8	801.7
Rondônia	120.6	14.9	2,879.60
Maranhão	127.6	14.4	2,270.80
Sergipe	135.6	15.9	7,856.90
Piauí	147.5	0	2,146.30
Rio de Janeiro	151.8	16.7	3,045.10
Paraíba	153.6	19.1	2,101.60
Acre	202.1	42.7	1,597.00
Pará	229.7	6.7	3,134.90
São Paulo	252.3	44.3	8,009.20
Alagoas	263.5	29.2	6,924.80
Pernambuco	282.8	19.1	7,277.30
Rio Grande do Sul	289.0	67.8	7,269.30
Minas Gerais	290.5	32.8	4,021.60
Ceará	318.8	8.2	4,697.90
Bahia	344.7	14.9	8,410.60
Roraima	351.1	48.2	1,555.00
Distrito Federal	376.4	17.9	2,750.40
Mato Grosso do Sul	397.6	26.6	6,365.00
Espírito Santo	404.0	27.6	12,091.40
Mato Grosso	417.8	50.5	5,475.00
Goiás	443.8	62.2	5,665.20
Rio Grande do Norte	453.6	67.8	6,661.00
Santa Catarina	554.5	81.0	9,337.40
Paraná	599.9	58.0	9,620.90
Amapá	628.4	0	4.423
Tocantins	752.4	106.2	19,699.80
BRAZIL	314,5	39.1	5.686,20

OHA: oral health assistant; OHT: oral health technician.

Poisson multiple regression analysis revealed a lower risk of occupational accidents with increasing age. Compared with the youngest age group (18–29 years), the 40–49 year (aRR = 0.87; 95% CI= 0.78–0.98) and the 50–59 year (aRR = 0.82; 95% CI = 0.70–0.96) age groups had significantly lower risk. Additionally, women were 1.40 times at higher risk (95% CI = 1.14–1.73) than men for accidents involving biological material during dental instrument cleaning. Professionals from the southern region were at 1.20 times higher risk (95% CI = 1.10–1.35) compared to those from the southeastern region. OHAs and OHTs had a significantly higher risk: OHAs showed a 10.22 times higher risk (95% CI = 8.70–12.00), and OHTs showed a 9.88 times higher risk (95% CI = 8.30–11.70) compared to dentists. Additionally, risk of accidents was 1.22 times higher (95% CI = 1.10–1.35) when biological material involved blood-containing fluids with blood and 1.22 times higher (95% CI = 1.10–2.35) for fluids other than serum, blood, or plasma. Occupational risk was also 1.15 times higher (95% CI = 1.02–1.28) for percutaneous exposure and 0.28 times lower (95% CI = 0.19–0.40) for oral or ocular mucosal exposure compared with accidents without these types of exposure. The risk increased further when the instrument involved was blades/lancets (aRR = 2.53; 95% CI = 2.19–2.92) or other instruments (glass or not informed, aRR = 3.39; 95% CI = 3.08–3.72) as compared to needles (Table 3).

**Table 3:** Risk factors for occupational accidents involving biological material during dental instrument cleaning by dental care professionals. Brazil, 2015-2020.

Variables		aRR	95%CI	β	p-value*
Age (years)					
	18–29	1.00			
	30–39	0.99	0.90–1.08	-0.010	0.819
	40–49	0.87	0.78–0.98	-0.136	0.018
	50–59	0.82	0.70–0.96	-0.196	0.011
	> 60	0.88	0.60–1.28	-0.131	0.499
Sex					
	Male	1.00			
	Female	1.40	1.14–1.73	0.340	0.001
Region					
	Southeast	1.00			
	Northeast	1.12	0.99–1.26	0.117	0.057
	Central	1.11	0.97–1.34	0.106	0.114
	South	1.20	1.10–1.35	0.190	< 0.001
	North	1.11	0.93–1.33	0.108	0.252
Professional education					
	Dentist	1.00			
	OHA	10.22	8.70–12.00	2.325	< 0.001
	OHT	9.88	8.30–11.70	2.290	< 0.001
Employment status					
	Others	1.00			
	Civil servant	0.89	0.78-1.00	-0.119	0.055
	EFC	0.99	0.88–1.11	-0.009	0.872
Types of exposure					
Percutaneous					
	No	1.00			
	Yes	1.15	1.02–1.28	0.135	0.018
Oral/Ocular mucosa					
	No	1.00			
	Yes	0.28	0.19–0.40	-1.270	< 0.001
Biological material					
	Serum/blood/plasma	1.00			
	Cavity fluids	1.24	0.74–2.00	0.214	0.414
	Blood-containing fluid	1.22	1.10–1.35	0.199	< 0.001
	Other fluids	1.22	1.10–1.35	0.203	< 0.001
Instrument					
	Needle	1.00			
	Blade	2.53	2.19–2.92	0.929	< 0.001
	Others	3.39	3.08–-3.72	1.219	< 0.001
Vaccination against HBV					
	Yes	1.00			
	No	1.03	0.90–1.18	0.027	0.716
Intercept: −4.500					

Poisson multiple regression model with robust variance adjusted for all variables in the table; β: regression coefficient; EFC: employee with a formal contract; 95%CI: confidence interval at 95%; Arr: adjusted relative risk; HBV: hepatitis B virus;

* Wald chi-square test. Model parameters: McFadden's R² = 0.233; Pearson goodness-of-fit χ^2^: 3964,704; p = 1.000; Wald χ^2^ test for model significance: χ^2^ = 2131,55; p < 0.001.

Because these analyses were based on secondary data, substantial information was missing data, as detailed in the tables.

## Discussion

This study assessed the epidemiology of OAIBM among dental care professionals in Brazil during dental instrument cleaning. An extensive national database was utilized, including data from all Brazilian states and regions, covering the period from January 2015 to July 2020. The database allowed us to estimate the incidence of accidents in specific locations, providing information that can guide local and regional prevention strategies. The incidence of occupational accidents averaged 314.5 cases per 100,000 dental care professionals. Therefore, the frequent handling of dental instruments, given their variety and complexity, inherently occupational risks among dental care professionals.

Dental care professionals have been identified as highly vulnerable to percutaneous and mucocutaneous exposure incidents (PMEIs) because of the intrinsic nature of their activities.^19,20^ In the present study, most PMEIs occurred among OHAs and OHTs, corroborating findings from previous research.^19,21–23^ These discrepant findings may be explained, at least partially, by differences in the training of OHAs and OHTs compared with that of dentists, given that dentists receive more extensive undergraduate training in biosafety, particularly in infection control and proper processing of dental instruments. Moreover, among OHAs and OHTs, OAIBM may be attributed to their roles in disassembling, cleaning, packaging, and sterilizing sharp instruments/materials, as well as assisting in procedures during which accidents may occur both during and after dental interventions.^24^ Studies have shown higher rates of PMEIs during cleaning procedures among OHAs and OHTs, ranging from 62.7% to 84.6%.^24,25^ Furthermore, a study evaluating OAIBM during the cleaning of reusable medical devices in nursing found similar patterns, with a predominance of OAIBM among nursing technicians and assistants.^26^

Younger individuals exhibited a higher incidence of PMEIs, consistent with findings from previous studies.^23,27^ PMEIs involving young dental care professionals may be associated with their inexperience and lack of technical skills, who often require longer periods to adapt to clinical activities and healthcare routines.^28^

An increased risk was observed when the contaminant involved blood-containing fluids. In such cases, incidents were more frequently caused by blades, leading to percutaneous injuries among dentists, OHAs, and, OHTs.^19,23,25,27,29^ Among dentists, constant manipulation of instruments within the restricted visual field of the oral cavity, combined with the use of sharp objects, rotary instruments, and ultrasonic devices, have been identified as factors that increase the risk of PMEIs.^30^ In studies evaluating the cleaning of critical reusable medical devices, the prevalence rates of accidents range from 15.0% to 29.3%.^22,31,32^

Data regarding types of exposure among dental care professionals reveal concerns about failure to use PPE, notwithstanding daily exposure to occupational risks. In our findings, gloves were not worn in 7.2% of PMEIs, consistent with similar findings reported in the literature.^33,34^ While this study did not specify glove type, previous research has indicated that 83% of dentists wear thick rubber gloves during instrument cleaning.^35^

Regression analysis revealed a higher risk of OAIBM in the southern region of Brazil among dental care professionals, likely due to the higher number of OAIBM notifications in that region.^13^ Conversely, regional disparities may also be influenced by factors such as insufficient investment in occupational health initiatives in healthcare settings and limited professional training promoted by health programs. Thus, further qualitative and quantitative studies are needed to investigate the factors influencing OAIBM during dental instrument cleaning by dental care professionals across different regions of Brazil.

In dental services, the cleaning of dental instruments is a technical responsibility of the dentist. Accordingly, the main contribution of this study, given the risk identified during the cleaning of dental instruments, is that dentists are accountable for implementing both managerial and educational strategies to ensure the safety of their dental care team. Dentists should ensure the availability of PPE, guide the staff in its correct use, enforce full compliance with PPE use, provide the necessary equipment, and train workers under their supervision on occupational health and safety, as established by Brazilian law, (regulatory standard no. 3236). Greater investment by dentists in automated cleaning, notably ultrasonic cleaners, is warranted, as these devices represent the gold standard for dental instrument cleaning^8,9^ and play a key role in reducing accidents involving biological material. Furthermore, automated cleaning is critical not only for occupational safety but also for enhancing the quality of cleaning processes, key steps in achieving effective sterilization by adequately reducing microbial load. Manual cleaning remains necessary for complex instrument features, such as serrations and joints. Therefore, investment in automated cleaning systems further enhances the safety of patients.

### Study limitations

This study presents some limitations. First, the study utilized secondary data from SINAN, which varied in terms of information completeness. Many variables had missing data, suggesting low quality of information, which may have affected the results. Second, the possibility of incorrect filling of notification forms by the reporting professionals cannot be ruled out, which may have contributed to information bias. Third, response bias may have occurred, as the professionals may have omitted some information about the accidents during their evaluations. Fourth, the form does not include essential variables that could help understand the epidemiology of OAIBM during dental instrument cleaning, such as the type of glove used at the time of the accident and the type of cleaning (manual or automated). Finally, the possibility of underreporting should be considered, especially because the data were collected during the COVID-19 pandemic. Consequently, incidence rates may have been underestimated, thus affecting the analyses of risk factors for OAIBM in this study.

## Conclusions

Despite the study limitations, the findings indicate that OAIBM during the dental instrument cleaning occurs throughout Brazil, affecting mainly younger OHAs and OHTs, with percutaneous injuries caused by blades. The findings also reveal negligence in the use of PPE. In this context, the study underscores the dentist's role as the leader and technical authority, responsible for providing adequate resources, standardizing procedures, training the staff, and supervising practices to promote occupational safety, proper instrument processing, and patient safety.

## Data Availability

The datasets used and/or analyzed in the present study are available from the corresponding author upon reasonable request.
